# Cytokine and Antibody Isotype Responses in Vaccinated Healthcare Workers with SARS-CoV-2 Breakthrough Infections

**DOI:** 10.3390/v17111517

**Published:** 2025-11-19

**Authors:** Miguel Ángel Fernández-Rojas, Tanya Plett-Torres, Guillermina Ávila, Mirza Romero-Valdovinos, Ana María Salazar, Monserrat Sordo, Mariana Chávez-Vargas, Cesar Josué Coeto Ángeles, Mayra Cruz-Rivera, Carlos Santiago-Olivares, Juan Pablo Ramírez Hinojosa, Pablo Maravilla, Patricia Ostrosky-Wegman, Fela Mendlovic, Ana Flisser

**Affiliations:** 1 Departamento de Microbiología y Parasitología, Facultad de Medicina, Universidad Nacional Autónoma de México (UNAM), Mexico City 04510, Mexico; 2 Plan de Estudios Combinados en Medicina, Facultad de Medicina, Universidad Nacional Autónoma de México (UNAM), Mexico City 04510, Mexico; 3 Hospital General “Dr. Manuel Gea González”, SSA. Calzada de Tlalpan 4800, Col. Seccion XVI, Tlalpan, Mexico City 14080, Mexico; 4 Departamento de Medicina Genómica y Toxicología Ambiental, Instituto de Investigaciones Biomédicas, Universidad Nacional Autónoma de México (UNAM), Mexico City 04510, Mexico; 5 Facultad de Ciencias de la Salud, Universidad Anáhuac México Norte, Huixquilucan 52786, State of Mexico, Mexico

**Keywords:** immunoglobulin isotypes, cytokines, BNT162b2 vaccine, healthcare workers, COVID-19

## Abstract

Background: Healthcare workers (HCWs) are at high risk of breakthrough SARS-CoV-2 infections despite complete vaccination schedules. There are gaps in our understanding of the specific antibody isotypes and cytokine profiles produced during an infection following vaccination. In this study, we evaluated SARS-CoV-2^−^specific antibody isotypes and their association with cytokine production in HCWs with breakthrough infections. Methods: Serum samples from 114 HCWs were analyzed for antibody isotypes against the nucleoprotein (NCP) and the receptor binding domain (RBD) of the spike protein, as well as for a panel of 13 cytokines. Results: Vaccinated SARS-CoV-2^+^ HCWs showed a higher prevalence of anti-SARS-CoV-2 antibodies against NCP (IgM = 93.8%, IgG = 93.8%, IgA = 28.1%) and RBD (IgM = 46.9%, IgG = 100%, IgA = 90.6%). A specific IgM response to NCP was more frequent in vaccinated SARS-CoV-2^+^ individuals, whereas IgA responses were predominantly specific for RBD. Both pro- and anti-inflammatory cytokines were elevated in vaccinated HCWs with breakthrough infections compared with unvaccinated and uninfected individuals. Interestingly, infected IgG+ HCWs with IgM specific for both NCP and RBD exhibited significantly higher IL-8, IL-6, TNF-α, IFN-γ, IL-2, IL-10, and TGF-β concentrations. Conclusion. Our data show that breakthrough infections in vaccinated HCWs induce a robust pro-and anti-inflammatory cytokine profile, which is associated with a broader IgM response directed against both NCP and RBD.

## 1. Introduction

Healthcare workers (HCWs) were among the groups at the highest risk of SARS-CoV-2 infection due to their continuous occupational exposure to the virus. This population was among the first to receive the BNT162b2 (BioNTech-Pfizer) vaccine during the first two waves of the pandemic in Mexico [1]. The BNT162b2 vaccine induces a robust cellular (T cell) and humoral (B cell) response in the naïve population and in individuals with a COVID-19 history. Infected and vaccinated individuals show elevated levels of IFN-γ and higher antibody neutralization activity [2,3]. Both full and incomplete vaccination regimens have been shown to be protective, increasing the time interval before reinfection with SARS-CoV-2, enhancing and sustaining specific antibody responses, and reducing the severity and mortality associated with COVID-19 [4,5].

Recent evidence has shown that mRNA vaccines trigger a transient anti-Spike (S)-IgM response coupled with robust anti-S IgG and IgA responses after the first dose. Enhanced anti-S IgG and IgA responses are observed after vaccine administration in patients who have previously been infected or exposed to the virus [6]. Additionally, a coordinated specific IgM/IgG response has been associated with a more efficient protective response [7]. IgM antibodies have been shown to be specific for SARS-CoV-2, while IgG and IgA have shown cross-reactivity with other coronaviruses [8,9].

Cytokines are involved in shaping the immune response during infection and after vaccination. In patients with COVID-19, higher levels of IL-1β, IL-6, and TNF-α are thought to contribute to inflammation and more severe outcomes, while IL-10 and TGF-β1 predominantly have regulatory effects, limiting excessive immune activation. A balanced cytokine response has been associated with better outcomes and suggested as a biomarker of an effective humoral response [10,11,12]. In contrast, after mRNA vaccination, studies usually describe a bias toward Th1-type cytokines, which support antiviral T-cell responses [13,14].

Although the immune response to SARS-CoV-2 vaccination in HCWs has been commonly studied [15,16], the relationship between antibody isotypes and cytokine profiles during breakthrough infection remains poorly defined. In this study, we evaluated the production of specific IgM, IgG, and IgA antibodies together with a panel of Th1-, Th2-, and Th17-associated cytokines, as well as regulatory and pro-inflammatory mediators, to characterize the relationship between humoral responses and systemic cytokine concentrations during breakthrough infection in this high-risk population.

## 2. Materials and Methods

### 2.1. Patient Information and Ethics Statement

This report is a retrospective cross-sectional analysis conducted among HCWs from the Hospital General Manuel Gea González, a public hospital in Mexico City. Samples were collected from December 2020 to March 2021. Demographic and clinical data were extracted from electronic health records, and each record was assigned a unique identification code to ensure confidentiality. All participants received two doses of the BNT162b2 (Pfizer- BioNTech) vaccine. Work areas were recorded for each participant (Supplementary Table S1). Participants reported cardiovascular comorbidities such as dyslipidemia, diabetes, hypertension, and overweight/obesity. The classification of overweight/obesity was determined according to the World Health Organization (WHO) standards for body mass index (BMI, kg/m^2^).

### 2.2. Ethical Statement

This study was approved by the Research and Ethics Committees of the Hospital General “Dr. Manuel Gea Gonzalez” (HGMGG), reference number 12-26-2020. Informed consent was obtained from all participants before enrollment. All procedures were conducted in accordance with the Declaration of Helsinki and the Federal Law on Personal Data Protection in Mexico (LFPDPPP).

### 2.3. Sample Processing

Blood samples were collected from all participants to obtain sera for antibody detection and cytokine quantification [17]. Saliva samples were collected for quantitative real-time-polymerase chain reaction (qRTPCR). Cycle threshold (Ct) values < 38 were interpreted as a positive result as previously described [18]. Briefly, 2 mL of saliva were collected from each HCW and frozen at −70 °C until use. Each 500 μL were supplemented with 10 µL of 2M dithiothreitol (DTT) and shaken for 30 min. Total RNA was extracted using TriPure isolation reagent according to the manufacturer’s instructions (Sigma-Aldrich, St. Louis, MO, USA) and eluted with 20 μL of Tris-EDTA buffer. Reverse transcription was carried out using oligo (dT) primers and GoScript Reverse Transcriptase (Promega, Madison, WI, USA). Real-time PCR was then performed with oligonucleotide primers specific to the SARS-CoV-2 Nucleocapsid protein (NCP). Primers for the human RNAse P were used as an internal control and saliva samples from healthy individuals served as negative controls.

### 2.4. Cytokine Assay

Cytokine concentrations (pg/mL) were measured using LEGENDplex Hu Essential Immune Response Panel (13-plex) (BioLegend, San Diego, CA, USA) according to the manufacturer’s instructions. This panel allows simultaneous quantification of Th1 (IFN-γ, IL-2, IL-12p70), Th2 (IL-4), and Th17 (IL-17A) cytokines, together with regulatory (IL-10, and TGF-β) and additional pro-inflammatory mediators (IL-1β, IL-6, TNF-α, IL-8, IP-10/CXCL10, MCP-1/CCL2).

### 2.5. SARS-CoV-2 Antibody Responses

To evaluate SARS-CoV-2 antibody responses (IgM, IgG, and IgA), we employed a recombinant SARS-CoV-2 Nucleocapsid protein expressed during the viral replication cycle (NCP, Cat. No. 40588-V08B) and a recombinant peptide corresponding to the receptor-binding domain (RBD, Cat. No. 40592-V08B) of the S protein from the original Wuhan strain that is in the outer part of the viral particle and against which all licensed vaccines have been designed (Sino Biological, Wayne, PA, USA) [19,20].

Specific IgM, IgG, and IgA antibody levels were measured in serum samples of HCWs using enzyme-linked immunosorbent assays (ELISAs). Serum was obtained by centrifugation (Thermo Scientific, Rockford, IL, USA) of whole blood at 800× *g* for 10 min at 4 °C and stored at −80 °C until use. Briefly, 100 µL of each antigen (0.5 µg/mL) was added per well on Inmunolon HB flat bottom plates (Thermo Scientific, Rockford, IL, USA) and incubated overnight at 4 °C. Plates were washed with PBS-Tween 0.1% and blocked with 200 µL of blocking solution (Thermo Scientific, Rockford IL, USA) for 1 h at room temperature. Subsequently, 100 µL of serum were added per well and incubated for 2 h at 37 °C. After washing, plates were incubated for 1 h at 37 °C with horseradish peroxidase (HRP)-conjugated anti-human antibodies: IgM (1:5000 dilution; Sigma-Aldrich, St. Louis, MO, USA), IgG (1:2000 dilution; Thermo Scientific, Rockford, IL, USA), and IgA (1:3000 dilution; Thermo Scientific, Rockford, IL, USA). Plates were then washed, and the reaction was stopped with 50 µL of 2N sulfuric. Absorbance was measured at 450 nm using an ELISA reader (iMak Bio-Rad, Hercules, CA, USA). Antibody positivity was obtained with an analysis of the ratio of optical density (OD) of HCW samples divided by the average OD of control samples [21].

Sensitivity and specificity for both antigens were evaluated in 86 samples, including 48 from uninfected controls without any respiratory disease collected between 2009 and 2011 and 38 samples from SARS-CoV-2-infected individuals collected between April and November 2020. A ROC curve analysis was performed using the OD values from HCW samples divided by the average OD of control samples. The cut-off values, along with the sensitivity and specificity for each antibody isotype were as follows: IgM (NCP = 1.76; 71%/100%, RBD = 1.8; 81%/100%), IgG (NCP and RBD = 2.4; 97%/100%), and IgA (NCP = 1.75; 89%/100%, RBD = 2.8; 97%/100%) (Supplementary Figure S1).

#### Data Handling and Statistical Analysis

Age was reported as the frequency of participants by deciles, except for older adults (≥60 years). The distributions of age and cytokine serum concentrations were expressed as median with interquartile ranges [IQR]. Frequencies of demographic variables (age, sex, and smoking status), clinical characteristics (diabetes, hypertension, obesity, SARS-CoV-2 infection, and vaccination status), and antibody seroprevalence were expressed as percentages. Differences between groups stratified by vaccination and infection status were analyzed using the Kruskal–Wallis test, as the Shapiro–Wilk test showed a non-normal distribution for age, cytokine concentrations, and OD values. We performed a multiple regression analysis to assess whether demographic and clinical variables were associated with infection and vaccination status and to evaluate potential cofounding effects. Our results showed no significant associations, so no adjustments were made in further analyses.

**Figure 1:**
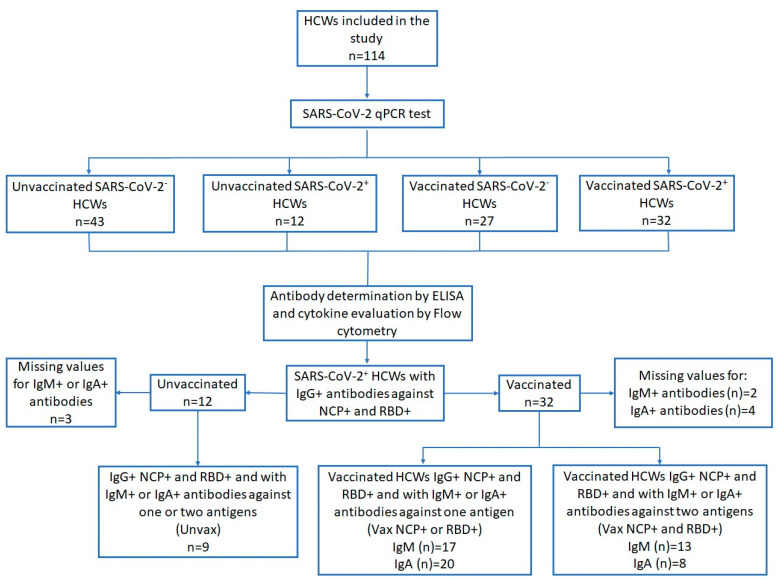
Selection diagram of SARS-CoV-2+ HCWs and of IgG^+^ individuals included in the evaluation of specific IgM and IgA responses in relation to cytokine concentrations.

Participants (*n* = 114) were classified into 4 groups according to their vaccination (vaccinated vs. unvaccinated) and infection status (SARS-CoV-2^+^ vs. SARS-CoV-2^−^). Demographic characteristics, SARS-CoV-2 antibody frequency, OD ratios, and cytokine concentrations were compared among these groups. To examine the association between the presence of specific IgM or IgA and cytokine concentrations, we classified the SARS-CoV-2^+^ individuals who were positive for anti-NCP and anti-RBD IgG according to their vaccination status. Participants were classified as follows: vaccinated HCWs with IgM or IgA antibodies against either NCP or RBD (Vax NCP^+^ or Vax RBD^+^) and vaccinated HCWs with IgM or IgA antibodies against both NCP and RBD (Vax NCP^+^ and RBD^+^). The unvaccinated and infected HCWs with specific IgM or IgA (Unvax) served as the reference group. This analysis can help distinguish the immune responses derived from vaccination (RBD-specific IgG/IgA), natural infection (NCP-specific IgG/IgM), and hybrid immunity (NCP- and RBD-specific IgG/IgM/IgA) (Figure 1). Differences in antibody frequencies were evaluated using Chi square or Fisher’s exact test as appropriate, and cytokine concentrations among these groups were evaluated using the Kruskal–Wallis test. Data analyses were performed using SPSS 23 (SPSS Inc., Chicago, IL, USA), while graphics were designed with GraphPad Prism 9 (GraphPad Software Inc., San Diego, CA, USA). We considered a *p* value ≤ 0.05 as a statistically significant threshold in all tests.

## 3. Results

### 3.1. Demographic and Clinical Characteristics of SARS-CoV-2^+^ HCWs

The study included 114 HCWs, 55 (48.2%) unvaccinated individuals (43 SARS-CoV-2^−^ and 12 SARS-CoV-2^+^) and 59 (51.7%) vaccinated with two doses of BNT162b2 (27 SARS-CoV-2^−^ and 32 SARS-CoV-2^+^). More than half of all participants were women (52.6%), younger than 40 years of age (58.2%), and without comorbidities (56.6%). Overall, 50% of participants had symptoms and 90.4% were non-smokers. The most frequent symptoms of SARS-CoV-2 infection were headache (42.1%) and myalgia (35.1%) (Table 1).

The prevalence of SARS-CoV-2 infection in the study population was 38.6% (44/114): 21.8% (12/55) in the unvaccinated group and 54.2% (32/59) among vaccinated individuals. All infections resulted in mild disease. Sex and age distribution was similar among the HCWs analyzed, regardless of their vaccination or infection status. All groups had a similar distribution of smokers. We found a higher but not significant frequency of comorbidities in unvaccinated and vaccinated SARS-CoV-2^+^ HCWs compared to SARS-CoV-2^−^individuals. As expected, unvaccinated and vaccinated HCWs who were SARS-CoV-2^+^ showed a higher frequency of symptoms (Table 1).

### 3.2. Anti-SARS-CoV-2 Specific Antibodies

**Figure 2:**
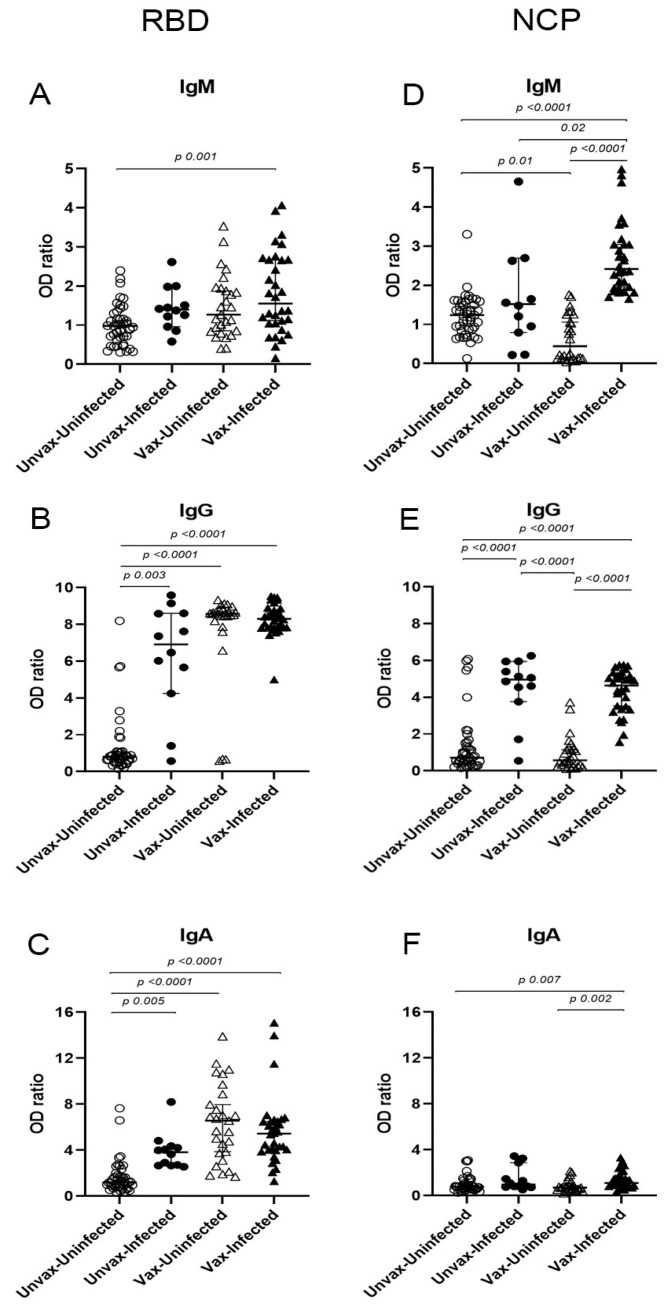
Anti-SARS-CoV-2 antibody isotype OD ratios in vaccinated and unvaccinated HCWs with or without SARS-CoV-2 infection. Serum OD ratios of IgM, IgG, and IgA specific to the receptor-binding domain of the spike protein (RBD) (**A**–**C**) and to the nucleoprotein (NCP) (**D**–**F**) were measured by ELISA. Graphs show the median OD ratios for each antibody isotype among HCWs classified by vaccination status (vaccinated, Vax; unvaccinated, Unvax) and infection status (SARS-CoV-2^−^, empty symbols; SARS-CoV-2^+^, filled symbols). *p* values were determined using the Kruskal–Wallis test. SARS-CoV-2: Severe acute respiratory syndrome coronavirus 2.

Figure 2 shows the isotype-specific responses against the RBD and NCP antigens. Vaccinated SARS-CoV-2^+^ HCWs exhibited a higher median OD ratio for IgM, IgG, and IgA antibodies compared with unvaccinated SARS-CoV-2^−^ individuals (Figure 2A–F). Within the vaccinated group, no significant differences were observed in RBD-specific isotype responses between SARS-CoV-2^+^ and SARS-CoV-2^−^ individuals. Vaccination alone (vaccinated SARS-CoV-2^−^) induced anti-RBD IgG and IgA, but not IgM. Furthermore, we did not observe statistical differences in anti-RBD isotype OD ratios between vaccinated SARS-CoV-2^+^ HCWs and their unvaccinated SARS-CoV-2^+^ counterparts (Figure 2A–C).

For the anti-NCP antibody response, vaccinated SARS-CoV-2^+^ HCWs exhibited significantly higher OD ratios for all three isotypes compared to SARS-CoV-2^−^ individuals. Among unvaccinated SARS-CoV-2^+^ HCWs, only anti-NCP IgG showed significantly elevated OD ratios relative to SARS-CoV-2^−^ individuals, with no differences observed for anti-NCP IgM or IgA. In contrast, vaccinated SARS-CoV-2^+^ HCWs had higher anti-NCP isotype responses than both SARS-CoV-2^−^ groups (Figure 2D–F). Interestingly, anti-NCP IgM OD ratios were significantly higher in vaccinated SARS-CoV-2^+^ individuals than in unvaccinated SARS-CoV-2^+^ individuals (Figure 2D). As expected, vaccination alone (vaccinated SARS-CoV-2^−^) did not elicit a specific anti-NCP antibody response, while natural infection alone (unvaccinated SARS-CoV-2^+^) induced significantly higher anti-NCP IgG OD ratios compared with vaccination alone (Figure 2E).

The prevalence of antibodies against NCP and RBD according to vaccination and infection status in HCW is shown in Table 2. Unvaccinated SARS-CoV-2^+^ HCWs (column A) showed significantly higher antibody prevalence than unvaccinated SARS-CoV-2^−^ individuals (column B), except for anti-RBD IgM and anti-NCP IgA. Unvaccinated SARS-CoV-2^−^ HCWs showed a low prevalence of all the isotypes against both antigens (column B). Anti-RBD IgG and IgA were highly prevalent in both vaccinated groups (C and D), while anti-RBD IgM was less frequent. Vaccinated SARS-CoV-2^+^ HCWs (column C) showed a significantly higher anti-NCP IgM prevalence compared to unvaccinated counterparts (column A). Vaccinated SARS-CoV-2^−^ HCWs (column D) also exhibited a higher prevalence of anti-RBD isotypes, and a modest but significant anti-NCP IgM response compared with the unvaccinated SARS-CoV-2^−^ group (column B), while no significant differences were seen for anti-NCP IgG or IgA.

### 3.3. Cytokine Levels in HCWs

**Figure 3:**
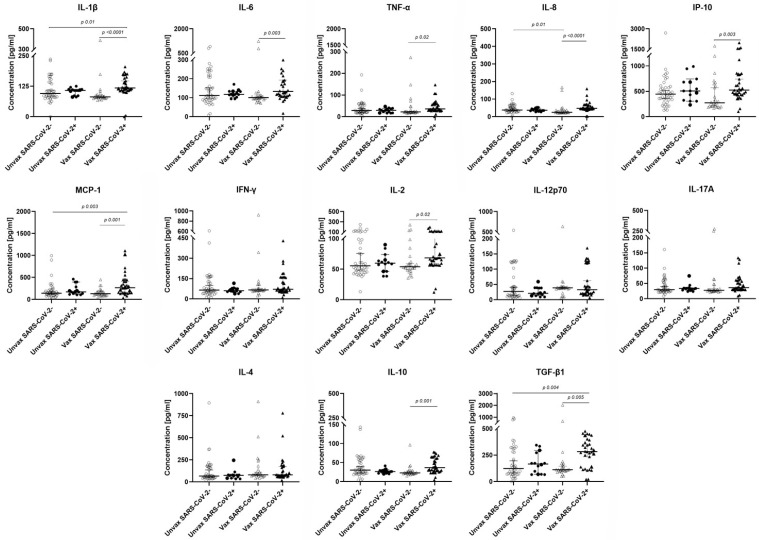
Cytokine concentrations in vaccinated and unvaccinated HCWs with or without SARS-CoV-2 infection. Serum cytokine levels were measured using the LEGENDplex Hu Essential Immune Response Panel. Graphs show median concentrations of each cytokine among HCWs classified by vaccination status (vaccinated, Vax; unvaccinated Unvax) and infection status (SARS-CoV-2^−^, empty symbols; SARS-CoV-2^+^, filled symbols). *p* values were determined using the Kruskal–Wallis test. SARS-CoV-2: Severe acute respiratory syndrome coronavirus 2.

Cytokine concentrations differed significantly based on the infection and vaccination status of the HCWs. Vaccinated SARS-CoV-2^+^ individuals exhibited a predominantly pro-inflammatory cytokine profile, with significantly higher concentrations of IL-1β, IL-6, TNF-α, IL-8, IP-10, MCP-1, and IL-2 compared with their vaccinated SARS-CoV-2^−^ counterparts. In addition, the anti-inflammatory cytokines (IL-10 and TGF-β) were elevated in vaccinated SARS-CoV-2^+^ individuals. When compared with the unvaccinated SARS-CoV-2^−^ group, the vaccinated SARS-CoV-2^+^ group had higher cytokine concentrations of IL-1β, MCP-1, and TGF-β (Figure 3).

### 3.4. Association of the IgM and IgA Responses with Cytokine Concentrations

To investigate the relationship between IgM and IgA seropositivity with cytokine responses in vaccinated HCWs, we analyzed IgG-positive individuals who were also positive for IgM or IgA antibodies against one or both antigens (Vax NCP^+^ or RBD^+^ and Vax NCP^+^ and RDB^+^, respectively). Unvaccinated HCWs served as the reference group (Unvax). Among vaccinated HCWs, 33.3% exhibited IgM antibodies against both NCP and RBD, while 43.7% were IgM-positive for a single antigen, all of which were specific to NCP (100%). For the IgA response, 21.7% of individuals were positive for both antigens, whereas positivity for a single antigen was predominantly directed against RBD (95%) (Supplementary Table S2).

**Figure 4:**
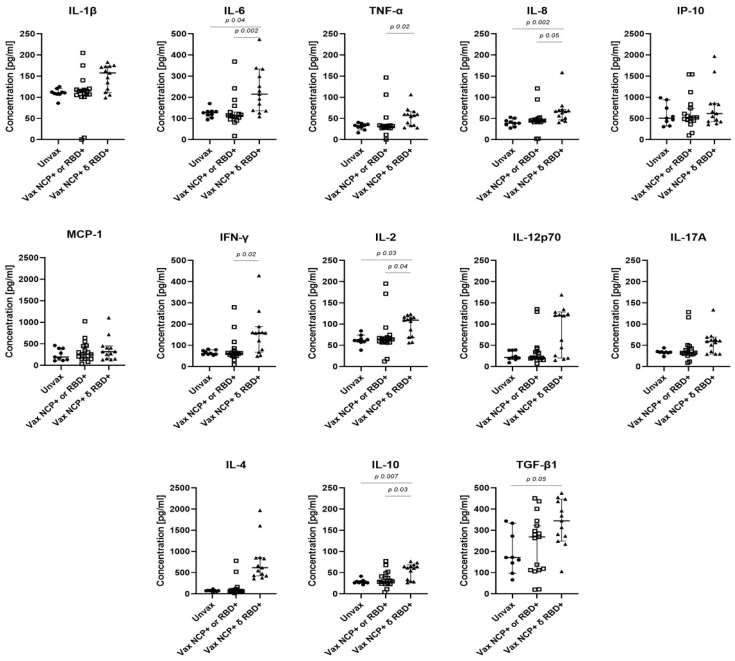
Serum cytokine concentrations in SARS-CoV-2-infected IgG+ HCWs according to their IgM seroprevalence. HWCs with IgG antibodies against NCP and RBD antigens (*n* = 39) were classified as Unvax (unvaccinated HWCs with IgM antibodies against NCP or RBD or both antigens), Vax NCP+ or RBD+ (vaccinated HCWs with IgM antibodies against either NCP or RBD) and Vax NCP+ and RBD+ (vaccinated HCWs with IgM antibodies against both antigens). Missing values = 4. Graphs show the median concentration of each cytokine. *p* values were determined using the Kruskal–Wallis test. SARS-CoV-2: Severe acute respiratory syndrome coronavirus 2.

Figure 4 shows the association between IgM responses and cytokine concentrations. Vaccinated HCWs who were seropositive for IgM antibodies against both viral antigens showed significantly higher concentrations of IL-6, IL-8, IL-2, IL-10, and TGF-β compared with the reference group. When compared with HCWs who had IgM responses restricted to NCP, significantly higher concentrations of IL-6, TNF-α, IL-8, IFN-γ, IL-2, and IL-10 were observed. A similar trend was observed for the IgA response, although differences did not reach statistical significance (Supplementary Figure S2).

## 4. Discussion

At the onset of the pandemic in Mexico, positive COVID-19 cases were underestimated, emphasizing the high exposure and impact of the pandemic in the country [22]. To date, few studies have examined the immune response in relation to the vaccination status of HCWs in Mexico and Latin America. Most research has predominantly focused on the IgG response, with relatively less attention to IgM and IgA. Studying the specific antibody isotypes and their association with cytokine levels in this population is important to inform effective containment measures for future outbreaks and improve public health interventions and next-generation vaccines.

Analyzing isotype-specific antibody responses provides a valuable tool for characterizing hybrid immunity that results from the combination of vaccination and natural infection. This approach helps to distinguish immune responses primarily associated with vaccination (RBD-specific IgG/IgA), natural infection (NCP-specific IgG/IgM), and hybrid immunity, which is characterized by stronger and broader antigenic coverage, (IgG/IgM/IgA directed against NCP and RBD).

Vaccinated SARS-CoV-2^+^ HCWs developed a mild anti-RBD IgM response, whereas their SARS-CoV-2^−^ counterparts did not exhibit detectable anti-RBD IgM. In contrast, vaccination induced strong anti-RBD IgG/IgA antibodies in both infected and uninfected individuals. These findings agree with studies showing that vaccination induces robust anti-RBD IgG/IgA but elicits minimal and short-lived IgM production [5,23]. Analyzing the IgM responses is relevant given its higher specificity for SARS-CoV-2, while IgG/IgA responses tend to exhibit greater cross-reactivity with other coronaviruses [8,9]. Furthermore, recent studies have shown that a coordinated anti-S IgM/IgG response after vaccination is associated with enhanced virus-neutralizing activity and a reduced risk of breakthrough SARS-CoV-2 infections, in contrast to vaccine recipients who develop a non-canonical IgG response in the absence of IgM [7,24]. Thus, the low anti-RBD IgM response found in our study population may in part explain the breakthrough infections observed, highlighting the need for specific isotype analyses.

When evaluating the antibody response to natural infection, we found that anti-NCP IgM levels were significantly higher in vaccinated individuals who tested positive for SARS-CoV-2, while the anti-NCP IgG levels were elevated in both unvaccinated and vaccinated SARS-CoV-2^+^ cases. These findings suggest that hybrid immunity can enhance early antibody responses to non-vaccine antigens such as NCP, while IgG against NCP remains a reliable marker of infection independent of vaccination status. Thus, vaccination and breakthrough infection results in an enhanced response from dual exposure to viral antigens. For example, in individuals vaccinated with mRNA vaccines (BNT162b2 or mRNA-1273) who subsequently experienced breakthrough infections with the Omicron variant, researchers found a robust humoral and cellular response characterized by a rapid release of anti-RBD IgG accompanied by an increase in memory T cells specific to non-spike antigens like NCP [25].

The stratification of vaccinated IgG+ individuals based on their IgM and IgA specificity to NCP and/or RBD revealed an association between dual IgM positivity (RBD + NCP) and elevated cytokine levels of IL-6, TNF-α, IL-8, IFN-γ, IL-2, IL-10, and TGF-β. This profile of acute-phase and regulatory cytokines supports the development of a more effective adaptive immune response. These findings reinforce the concept of hybrid immunity, which results from the combined immune response induced by vaccination and breakthrough infection. Although inefficient in preventing infection, hybrid immunity is associated with enhanced immune activation and protection against severe disease. This suggestion relates to the WHO’s interim statement, which recognized hybrid immunity as contributing to stronger and broader protection against SARS-CoV-2 [26]. With the high prevalence of SARS-CoV-2 breakthrough infections, hybrid immunity is becoming more common [27]. Our results demonstrate distinct isotype-specific antibody profiles and elevated cytokine responses in vaccinated HCWs with breakthrough infections, highlighting the relevance of hybrid immunity in shaping the immune response to COVID-19.

Regarding the anti-NCP IgA response, vaccinated individuals with SARS-CoV-2 infection showed a minimal increase in IgA levels. This observation is consistent with recent studies indicating that the anti-NCP IgA response is low and does not significantly vary with vaccination or infection status [28]. This may explain why we did not observe an association of IgA with cytokine levels, although we found a similar trend to that of IgM, where individuals with dual antigen positivity (RBD and NCP) tended to show elevated cytokine concentrations.

Our data demonstrate that both pro- and anti-inflammatory cytokine concentrations are elevated in vaccinated SARS-CoV-2^+^ HCWs. The pro-inflammatory cytokines IL-8, IL-1β, IL-6, TNF-α, IFN-γ, and IL-2 are mediators of neutrophil recruitment, acute inflammation and T cell activation [29]. These findings suggest the activation of both innate and adaptive immunity in these individuals. The pro-inflammatory profile was accompanied by the anti-inflammatory cytokines IL-10 and TGB-β, known for their regulatory role to control inflammation and promote resolution [12,30]. These data suggest a balanced immune regulation that may contribute to the mild disease outcomes observed in all infected HCWs. Previous studies have shown that vaccination with the BNT162b2 vaccine induces an increment of chemokines and cytokines such as IP-10, IFN-γ, TNF-α, IL-15, and IL-10 in SARS-CoV-2^+^ vaccinated individuals [14,31]. Moreover, a specific cytokine profile, including IL-2, TNF-α, IL-9, and IP10, produced by CD4 T cells has been identified as a suitable signature to predict the activation and functional response of CD8 T cells after vaccination following BNT162b2 vaccination [32]. Breakthrough infections are often associated with a more balanced cytokine environment and mild disease outcomes, as well as enhanced T cell responses and epitope repertoires [33].

Some limitations of our study include the relatively small sample size, recruitment from a single public hospital, the convenience-based sample selection due to difficulties in participant enrollment during confinement measures, the absence of detailed records on infection dates, and the descriptive nature of the study inherent to its cross-sectional design. Therefore, longitudinal follow-up studies in HCWs, as well as retrospective and multicenter studies, are needed to provide insights on long-term immunity, which could help refine booster vaccination schedules, particularly in highly exposed individuals. Nevertheless, our study provides novel insights into the association of the specific IgM response with the cytokine profile in HCWs, an aspect that has not been comprehensively addressed during the years of high COVID-19 prevalence in Mexico.

## 5. Conclusions

Our results support the concept of hybrid immunity resulting from the combination of vaccine-induced and infection-acquired immunity. Dual seropositivity to NCP and RBD antigens together with elevated pro- and anti-inflammatory cytokines, suggests that a coordinated IgM response may contribute to a more functional, rapid, and regulated immune activation following breakthrough infection in vaccinated HCWs. Given that IgM antibodies are generally more specific for SARS-CoV-2, compared with the more cross-reactive IgG and IgA, characterizing the dynamics and specificity of isotype responses, particularly IgM, in parallel with cytokine profiles could provide valuable insights into recent viral exposure, the quality of the immune response, and may aid in designing tailored vaccination strategies.

## Figures and Tables

**Table 1:** Demographic characteristics of the study participants.

Characteristic *n* = 114 (n; %)	Unvaccinated SARS-CoV-2^−^ *n* = 43 n (%; 95% CI)	Unvaccinated SARS-CoV-2^+^ *n* = 12 n (%; 95% CI)	Vaccinated SARS-CoV-2^−^ *n* = 27 n (%; 95% CI)	Vaccinated SARS-CoV-2^+^ *n* = 32 n (%; 95% CI)	*p* Value
Sex Female (60; 52.6) Male (54; 47.4)	26 (60.5; 45.2–75.7) 17 (39.5; 24.3–54.8)	4 (33.3; 2.05–64.6) 8 (66.3; 35.4–97.9)	17 (63.0; 43.5–82.4) 10 (37.0; 17.6–56.5)	13 (40.6; 22.6–58.6) 19 (59.4; 41.4–77.4)	0.121
Age Groups ^a^ 20–29 (29; 29.6) 30–39 (28; 28.6) 40–49 (11; 11.2) 50–59 (20; 20.4) ≥60 (10; 10.2)	7 (20.6; 6.3–34.9) 14 (41.2; 23.7–58.6) 2 (5.9; −2.4–14.2) 7 (20.6; 6.3–34.9) 4 (11.8; 0.3–23.2)	3 (27.3; −4.1–58.6) 4 (36.4; 2.5–70.3) 2 (18.8; −8.9–45.4) 2 (18.8; −8.9–45.4) 0	9 (33.3; 14.3–52.3) 4 (14.8; 5.0–29.1) 2 (7.4; −3.1–17.9) 7 (25.9; 8.3–43.6) 2 (7.4; −3.1–17.9)	10 (31.2; 14.3–48.2) 6 (18.8; 4.5–33.1) 5 (15.6; 2.3–28.9) 4 (12.5; 4.0–24.6) 4 (12.5; 4.0–24.6)	0.497 0.141 0.434 0.587 0.610
Comorbidities ^b^ No (56; 56.6) Yes (43; 43.4) Types ^c^ Overweight/Obesity (42; 97.7) Hypertension (3; 7.0) Dyslipidemia (1; 2.3)	21 (63.6; 46.3–80.9) 12 (36.4; 19.0–53.7) 12 (36.4; 19.0–53.7) 0 0	4 (57.1; 7.7–106.6) 3 (42.9; 6.6–92.3) 3 (42.9; −6.6–92.3) 0 0	17 (63.0; 43.5–82.4) 10 (37.0; 17.6–56.5) 10 (37.0; 17.6–56.5) 1 (3.7; −3.9–11.3) 1 (3.7; −3.9–11.3)	14 (43.7; 25.6–61.9) 18 (56.3; 38.1–74.4) 17 (53.1; 34.8–71.4) 2 (6.3; −2.6–15.1) 0	0.358
Smoking No (103; 90.4) Yes (11; 9.6)	40 (93.0; 85.1–100.9) 3 (7.0; −0.9–14.9)	11 (91.7; 73.3–110) 1 (8.3; −10.0–26.7)	24 (88.9; 76.2–101.6) 3 (11.1; −1.6–23.8)	28 (87.5; 75.4–99.6) 4 (12.5; 3.9–24.6)	0.865
Symptoms Asymptomatic (57; 50.0) Symptomatic (57; 50.0) Fever (25; 21.9) Myalgia (40; 35.1) Anosmia (22; 19.3) Rhinorrhea (28; 24.6) Diarrhea (14; 12.3) Fatigue (22; 19.3) Erythema (2; 1.8) Odynophagia (31; 27.2) Headache (48; 42.1) Dysgeusia (22; 19.3)	36 (83.7; 72.2–95.2) 7 (16.3; 4.8–27.8) 2 (4.6; −1.9–11.2) 1 (2.3; −2.4–7.0) 1 (2.3; −2.4–7.0) 6 (13.9; 3.2–24.7) 2 (4.6; −1.9–11.2) 6 (13.9; 3.2–24.7) 0 3 (6.9; −0.9–14.1) 6 (13.9; 3.2–24.7) 0	2 (16.7; −8.1–41.4) 10 (83.3; 58.6–108.1) 6 (50.0; 16.8–83.2) 9 (75.0; 46.3–103.7) 7 (58.3; 25.6–91.1) 2 (16.7; −8.1–41.4) 2 (16.7; −8.1–41.4) 4 (33.3; 2.1–64.6) 1 (8.3; −10.0–26.7) 4 (33.3; 2.1–64.6) 7 (58.3; 25.6–91.1) 7 (58.3; 25.6–91.1)	16 (59.3; 39.5–79.1) 11 (40.7; 20.9–60.5) 1 (3.6; −3.8–10.9) 5 (17.9; 2.7–32.9) 0 5 (17.9; 2.7–32.9) 1 (3.6; −3.8–10.9) 7 (25.0; 7.9–42.1) 0 3 (10.7; −1.5–22.9) 7 (25.0; 7.9–42.1) 1 (3.6; −3.8–10.9)	3 (9.4; −1.3–20.0) 29 (90.6; 79.9–101.3) 16 (50.0; 31–7−68.3) 25 (78.1; 62.9–93.3) 14 (43.8; 25.6–61.9) 15 (46.9; 28.6–65.1) 9 (28.1; 11.6–44.6) 5 (12.8; 2.3–28.9) 1 (2.6; −3.2–9.5) 21 (65.6; 48.2–83.0) 28 (87.5; 75.4–99.6) 14 (43.8; 25.6–61.9)	0.0001 0.0001 0.0001 0.0001 0.007 0.008 0.345 0.206 0.0001 0.0001 0.0001

Bold values indicate statistically significant values. ^a^ Missing values = 16: 9 in Unvaccinated SARS-CoV-2^−^, 1 in Unvaccinated SARS-CoV-2^+^, 3 in Vaccinated SARS-CoV-2^−^ and 3 in Vaccinated SARS-CoV-2^+^; ^b^ Missing values = 15: 10 in Unvaccinated SARS-CoV-2^−^, 5 in Unvaccinated SARS-CoV-2^+^; ^c^ It should be noted that some categories can be counted more than once. SARS-CoV-2 prevalence: SARS-CoV-2^+^ 44/114 = 38.6% and SARS-CoV-2^−^ 70/114 = 61.4%. SARS-CoV-2: Severe acute respiratory syndrome coronavirus 2. *p* values for Kruskal–Wallis test.

**Table 2:** Prevalence of anti-SARS-CoV-2 antibodies in HCWs.

Antibody	Antigen	A Unvaccinated SARS-CoV-2^+^ *n* = 12 (%)	B Unvaccinated SARS-CoV-2^−^ *n* = 43 (%)	C Vaccinated SARS-CoV-2^+^ *n* = 32 (%)	D Vaccinated SARS-CoV-2^−^ *n* = 27 (%)	*p* Value			
						A vs. B	A vs. C	C vs. D	B vs. D
IgM	NCP	4 (33.3)	2 (4.7)	30(93.8)	1 (3.7)	0.017	0.0001	0.0001	1.000
	RBD	3 (25.0)	3 (7.0)	15 (46.9)	9 (33.3)	0.110	0.303	0.425	0.008
IgG	NCP	10 (83.3)	5 (11.6)	30 (93.8)	2 (7.4)	0.0001	0.297	0.0001	0.699
	RBD	10 (83.3)	5 (11.6)	32 (100)	24 (88.9)	0.0001	0.070	0.090	0.0001
IgA	NCP	3 (25.0)	4 (9.3)	9 (28.1)	3 (11.1)	0.168	1.000	0.193	1.000
	RBD	8 (66.7)	4 (9.3)	29 (90.6)	22 (81.5)	0.0001	0.075	0.450	0.0001

HCWs: healthcare workers; NCP: Nucleoprotein; RBD: Receptor-binding domain. SARS-CoV-2: Severe acute respiratory syndrome coronavirus 2. Each *p* value for Fisher’s exact test.

## Data Availability

All data have been reported in this manuscript.

## Supplementary Materials

The following supporting information can be downloaded at: https://www.mdpi.com/article/10.3390/v17111517/s1, Figure S1: ROC curve analysis of IgM, IgG and IgA antibodies against SARS-CoV-2 proteins NCP and RBD; Figure S2: Comparison of the concentration of serum cytokines among SARS-CoV-2^+^ HCWs according to their IgA antibody seroprevalence; Table S1: Distribution of Healthcare workers based on their working areas; Table S2: Frequency of IgM and IgA response against NCP and/or RBD antigens in IgG^+^ SARS-CoV-2^+^ HCWs.
