# Supplementary material for: Cytokine and Antibody Isotype Responses in Vaccinated Healthcare Workers with SARS-CoV-2 Breakthrough Infections
Source: Viruses. 2025 Nov 19;17(11):1517. doi: 10.3390/v17111517 (PMC12656851; doi:10.3390/v17111517)
Supplement: Supplementary file 1 [file viruses-17-01517-s001.zip › viruses-3934812-supplementary.pdf]

Supplementary Materials

Cytokine and Antibody Isotype Responses in Vaccinated Healthcare Workers with SARS-CoV-2 Breakthrough Infections

Table S1. Distribution of Healthcare workers based on their working areas.

Job (n= 114)	n (%)
Nurses	6 (5.3)
Physicians	76 (66.7)
Researchers/Students	15 (13.1)
Secretaries	9 (7.9)
Laboratory technicians	8 (7.0)

Table S2. Frequency of IgM and IgA response against NCP and/or RBD antigens in IgG^+ SARS-CoV-2^+ HCWs.

	IgG^+ NCP^+ and RBD^+ n=39	
**Antigen response**	**IgM[a]** ***n* = 39 (%)**	**IgA[a]** ***n* = 37 (%)**
Unvax	9 (23)	9 (24.3)
Vax NCP or RBD	17 (43.7)	20 (54)
Vax NCP	17 (100)	1 (5)
Vax RBD	---	19 (95)
Vax NCP & RBD	13 (33.3)	8 (21.7)

HCWs: Health care workers; NCP: Nucleoprotein; RBD: Receptor-binding domain. SARS-CoV-2: Severe acute respiratory syndrome coronavirus 2. [a]Missing data= 2 because the HCW do not develop IgA antibodies.

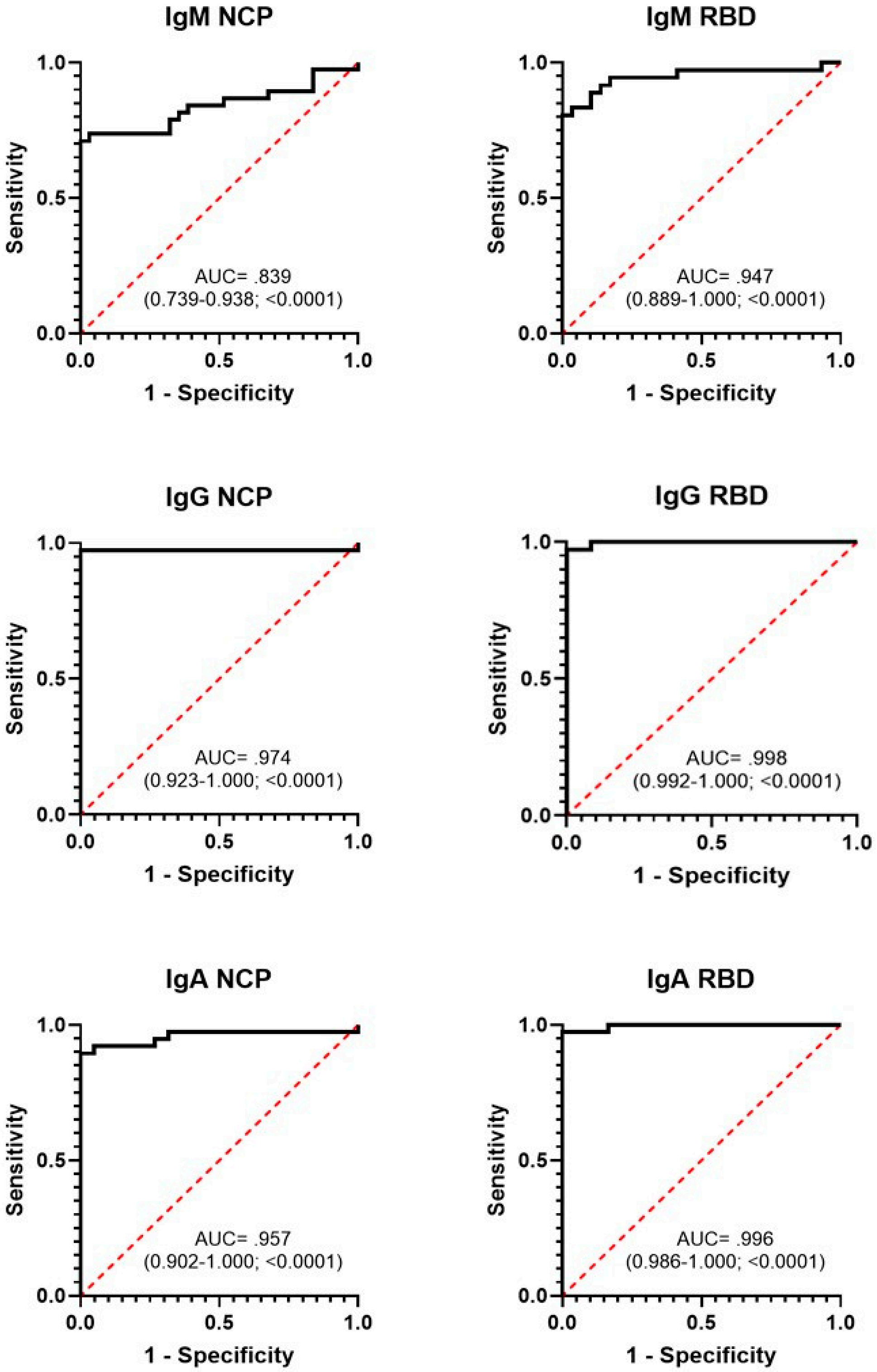

Figure S1. ROC curve analysis of IgM, IgG and IgA antibodies against SARS-CoV-2 proteins NCP and RBD. Each graph shows their area under the curve (AUC) and the 95% confidence interval and *p* value. Missing data: IgM (NCP=19 uninfected; RBD= 19 uninfected, 1 infected), IgG (NCP=3 uninfected; RBD= 2 infected) and IgA (NCP=7 uninfected; RBD= 12 uninfected).

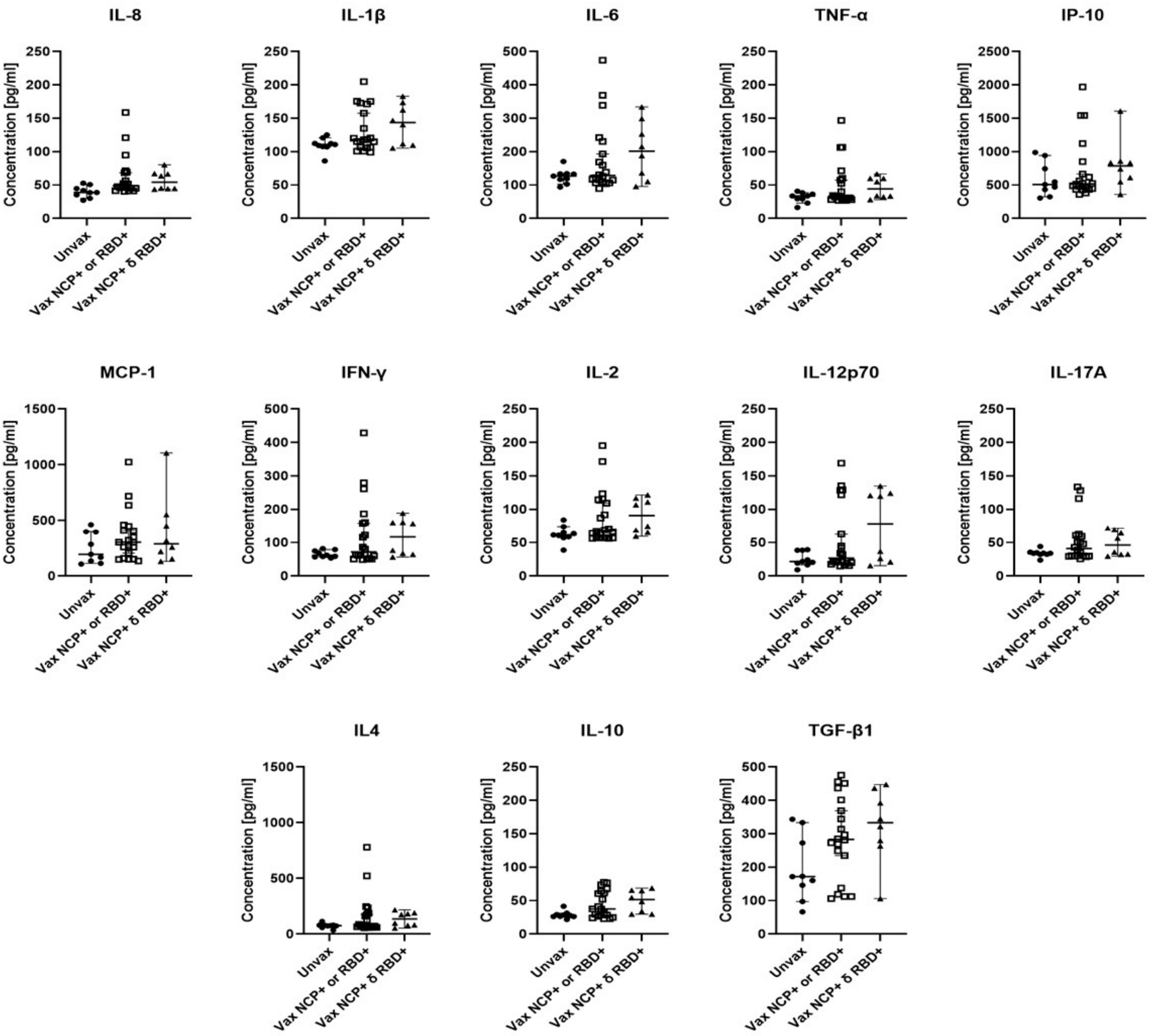

Figure S2. Comparison of the concentration of serum cytokines among SARS-CoV-2+ HCWs according to their IgA antibody seroprevalence. Cases correspond to HCWs with IgG antibodies against NCP and RBD antigens (n=39) that were classified as Unvax (unvaccinated HWCs with IgA antibodies against NCP or RBD or both antigens), Vax NCP+ or RBD+ (vaccinated HCWs with IgA antibodies against NCP or RBD) and Vax NCP+ and RBD+ (vaccinated HCWs with IgA antibodies against both antigens). Graphs show the median concentration of each cytokine. SARS-CoV-2: Severe acute respiratory syndrome coronavirus 2. *p* values for the Kruskal-Wallis test.
